# Storage Stability of 6FDA-DMB Polyamic Acid Solution Detected by Gel Permeation Chromatography Coupled with Multiple Detectors

**DOI:** 10.3390/polym15061360

**Published:** 2023-03-09

**Authors:** Mei Hong, Wei Liu, Runxiang Gao, Rui Li, Yonggang Liu, Xuemin Dai, Yu Kang, Xuepeng Qiu, Yanxiong Pan, Xiangling Ji

**Affiliations:** 1 State Key Laboratory of Polymer Physics and Chemistry, Changchun Institute of Applied Chemistry, Chinese Academy of Sciences, Changchun 130022, China; 2 School of Applied Chemistry and Engineering, University of Science and Technology of China, Hefei 230026, China; 3 CAS Key Laboratory of High-Performance Synthetic Rubber and Its Composite Materials, Changchun Institute of Applied Chemistry, Chinese Academy of Sciences, Changchun 130022, China

**Keywords:** polyamic acid, molecular weight, storage condition, degradation, hydrolysis

## Abstract

Polyamic acid (PAA) is the precursor of polyimide (PI), and its solution’s properties have a direct influence on the final performances of PI resins, films, or fibers. The viscosity loss of a PAA solution over time is notorious. A stability evaluation and revelation of the degradation mechanism of PAA in a solution based on variations of molecular parameters other than viscosity with storage time is necessary. In this study, a PAA solution was prepared through the polycondensation of 4,4′-(hexafluoroisopropene) diphthalic anhydride (6FDA) and 4,4′-diamino-2,2′-dimethylbiphenyl (DMB) in DMAc. The stability of a PAA solution stored at different temperatures (−18, −12, 4, and 25 °C) and different concentrations (12 wt% and 0.15 wt%) was systematically investigated by measuring the molecular parameters, including *M*_w_, *M*_n_, *M*_w_/*M*_n_, *R*_g_, and [*η*], using gel permeation chromatography coupled with multiple detectors (GPC-RI-MALLS-VIS) in a mobile phase 0.02 *M* LiBr/0.20 *M* HAc/DMF. The stability of PAA in a concentrated solution decreased, as shown by the reduction ratio of *M*_w_ from 0%, 7.2%, and 34.7% to 83.8% and that of *M*_n_ from 0%, 4.7%, and 30.0% to 82.4% with an increase of temperature from −18, −12, and 4 to 25 °C, respectively, after storage for 139 days. The hydrolysis of PAA in a concentrated solution was accelerated at high temperatures. Notably, at 25 °C, the diluted solution was much less stable than the concentrated one and exhibited an almost linear degradation rate within 10 h. The *M*_w_ and *M*_n_ decreased rapidly by 52.8% and 48.7%, respectively, within 10 h. Such faster degradation was caused by a greater water ratio and less entanglement of chains in the diluted solution. The degradation of (6FDA-DMB) PAA in this study did not follow the chain length equilibration mechanism reported in literature, given that both *M*_w_ and *M*_n_ declined simultaneously during storage.

## 1. Introduction

Polyimide (PI) is one of the high-performance polymers with merits, including high-temperature resistance, high mechanical strength, and excellent electrical properties, and is widely applied in aerospace, electronics, electrical, and other high-tech fields [1,2]. Usually, dianhydride and diamine are polycondensed in polar aprotic solvent such as *N*,*N*-dimethylformamide (DMF), *N*,*N*-dimethylacetamide (DMAc), or *N*-methyl-2-pyrrolidone (NMP) to produce the precursor of PI: polyamic acid (PAA), which transforms to PI through thermal or chemical imidization.

Indeed, the properties of a PAA solution play a decisive role in the final performance of the PI products [3,4]. However, PAA solutions are relatively unstable upon storage and exhibit undesirable variations in viscosity with different storage conditions [5,6,7,8,9,10,11,12]. Bower and Frost [5] observed a viscosity reduction of 11% DAPE-PMDA in DMAC with increasing temperature. Sroog et al. [6] investigated the effects of concentration and added water on the viscosity of a (POP-PA) PAA solution in DMAc stored at 23 °C for 30 days using a viscometer and noted a decrease in viscosity with added water. Frost and Kesse [13] also found that the viscosity dropped much more rapidly by increasing the water content in a (PMDA-MDA) PAA solution. They ascribed this viscosity deterioration of the PAA solution to degradation by hydrolysis. Walker [14] studied the variation in both the weight-average molecular weight (*M*_w_) and number-average molecular weight (*M*_n_) of freshly prepared (PMDA-ODA) PAA solutions at 31 and 8 °C through gel permeation chromatography (GPC) using 0.03 *M* LiBr/0.03 *M* H_3_PO_4_/1 vol% THF/DMF as a mobile phase. The results indicated a reduction in *M*_w_ and almost constant *M*_n_. The author considered that the viscosity decrease of a freshly prepared PAA solution was related to chain length equilibration rather than degradation of PAA, and the crucial parameter in determining the hydrolytic degradation of PAA was only the decrease of *M*_n_.

Previous studies have given some explanations for the viscosity reduction of PAA solution upon storage. One explanation is the hydrolytic degradation of PAA with trace water in the solvent [6,9,10]. Another interpretation is that PAA has chain length equilibration through a reversible polyamic acid-forming reaction [14,15,16]. Recently, Zhang et al. [17] proposed that the chain conformation arrangement of PAA during storage may also account for the viscosity decrease. However, only using viscosity to investigate and evaluate the stability of PAA solution is limited and cannot fully explain the mechanism of PAA degradation. Determining molecular weights, including *M*_w_, *M*_n_, and other molecular parameters, is necessary to explore the essence of PAA instability. PAA as a polyelectrolyte generally caused anomalous GPC elution behaviors, such as adsorption to the columns, earlier elution, and so on [15,18,19]. Therefore, precise characterization of the molecular weights and other molecular parameters of PAA is always tricky but highly desired for understanding and controlling the solution properties of PAA. Our previous study [20] reported an efficient, mild, and universal GPC mobile phase 0.02 *M* LiBr/0.20 *M* HAc/DMF for successfully measuring the accurate molecular parameters of PAA by shielding complicated interactions in the solution. This work aimed to further systematically study the variations of molecular weights and other molecular parameters of PAA during storage. In this study, we used polyamic acid formed from the polycondensation of 4,4′-(hexafluoroisopropene) diphthalic anhydride (6FDA) and 4,4′-diamino-2,2′-dimethylbiphenyl (DMB) to detect the changes of molecular parameters, including *M*_w_, *M*_n_, *M*_w_/*M*_n_, root-mean-square radius of gyration (*R*_g_), and intrinsic viscosity ([*η*]), using GPC coupled with multiple detectors (GPC-RI-MALLS-VIS) under different storage temperatures and times. These results will definitely provide suggestions on tuning PAA solution properties and further control the related performances of PI materials.

## 2. Materials and Methods

### 2.1. Materials

We purchased 6FDA and DMB from Beijing Multi Technology Co., Ltd. (Beijing, China) The 6FDA was purified through sublimation before use. *N*,*N*′-Dimethylacetamide (DMAc, AR) purchased from Beijing Chemical Works (Beijing, China) was dried over P_2_O_5_ and used as the solvent for polycondensation. *N*,*N*′-Dimethylformamide (DMF, HPLC) was purchased from Tedia Co., Ltd. (Fairfield, CT, USA). Anhydrous lithium bromide (LiBr, 99.99%) supplied by Alfa Aesar was dried at 130 °C for 8 h under vacuum before use. Acetic acid (HAc, 99.7%) was purchased from Sigma-Aldrich (Shanghai, China).

### 2.2. Preparation of (6FDA-DMB) PAA Concentrated Solution

**Scheme 1:**
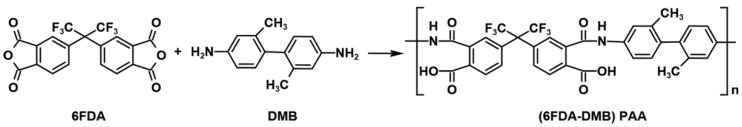
Formation of (6FDA-DMB) PAA through polycondensation.

The synthesis of (6FDA-DMB) PAA was the same as our previous report [20]. Specifically, DMB was first dissolved in DMAc by stirring at room temperature under a nitrogen atmosphere. Next, stoichiometric amounts of 6FDA were added to react with DMB at 20 °C under a nitrogen atmosphere for 48 h to achieve a (6FDA-DMB) PAA solution with a concentration of 12 wt%. The solution was sealed and stored in the refrigerator at −18 °C. The formation of (6FDA-DMB) PAA is displayed in Scheme 1.

### 2.3. Storage Conditions of (6FDA-DMB) PAA Concentrated and Diluted Solution

The concentrated solution of (6FDA-DMB) PAA was sealed in brown bottles and stored at different temperatures (−18 °C, −12 °C, 4 °C, and 25 °C) with a fluctuation range of ±1 °C for different days (0, 2, 10, 15, 30, and 139 days). Samples were taken out at various intervals for a GPC-RI-MALLS-VIS measurement.

The diluted solution of (6FDA-DMB) PAA was obtained by diluting the concentrated one to 0.15% and subsequently sealing it in brown bottles and storing it at 25 °C for different hours (0, 1, 3, 6, and 10 h). Samples were taken out periodically for a GPC-RI-MALLS-VIS measurement.

### 2.4. GPC-RI-MALLS-VIS Characterization

The GPC-RI-MALLS-VIS system includes a Waters 515 pump; Waters 717 plus autosampler; Waters 2414 differential refractive index (RI) detector; DAWN HELEOS II eighteen-angle laser light scattering (MALLS) detector (Wyatt Technologies, Santa Barbara, USA), including 22.5°, 28.0°, 32.0°, 38.0°, 44.0°, 50.0°, 57.0°, 64.0°, 72.0°, 81.0°, 90.0°, 99°, 108°, 117.0°, 126.0°, 134.0°, 141.0°, and 147.0°, where 99° is a dynamic light scattering (DLS) detector; ViscoStar viscometer (VIS, Wyatt Technologies); and two PLgel 10 μm MIXED-B LS columns (300 × 7.5 mm, Agilent Technologies, Beijing, China). The temperature of the columns and three detectors were 35 °C. The mobile phase was 0.02 *M* LiBr/0.20 *M* HAc/DMF. The flow rate was 1.0 mL/min, and the injection volume was 100 μL. Pure toluene of HPLC purity was used for calibration of the light-scattering photometer. The normalization coefficient of the MALLS detector was obtained using polystyrene narrow standards with a molecular weight of 19,000 g/mol. The calibration constant of the RI detector was determined also by this polystyrene narrow standard. The volume delay between the MALLS and RI detectors and the MALLS and VIS detectors were determined using the polystyrene narrow standard. The Berry method [21] with a linear fit was applied to the MALLS data processing.

All samples for GPC measurements were dissolved in the mobile phase, with a concentration of about 1.5 mg/mL, and filtered through 0.20 μm or 0.45 μm PTFE membranes.

## 3. Results and Discussion

### 3.1. Molecular Parameter Characterization of (6FDA-DMB) PAA

**Figure 1:**
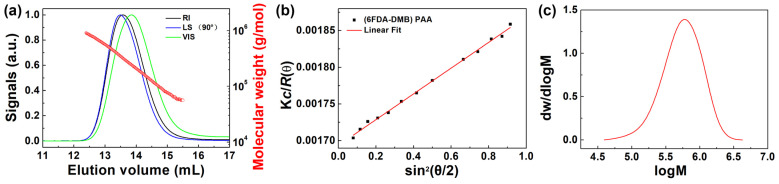
(**a**) RI, LS (90°), VIS signals, and logarithm of molecular weight as a function of elution volume. (**b**) *Kc*/*R*(*θ*) vs. sin^2^(*θ*/2) plot. (**c**) Molecular weight distribution curve for original (6FDA-DMB) PAA.

The optimal mobile phase for a GPC measurement of PAA is 0.02 *M* LiBr/0.20 *M* HAc/DMF, as determined by our group in a preliminary study [20]. The d*n*/d*c* of (6FDA-DMB) PAA in this mobile phase is 0.145 mL/g. The multi-detector elution curves of (6FDA-DMB) PAA are shown in Figure 1. As can be seen from Figure 1a, all the elution curves measured by the refractive index (RI) detector, light scattering (LS) at 90° detector, and viscometer (VIS) were symmetrical and unimodal. In addition, the logarithm of molecular weight and the elution volume had a good linear relationship over a wide range of elution volume. Figure 1b shows the variation of scattered light intensity *Kc*/*R*(*θ*) with scattering angles sin^2^(*θ*/2). The data points from the lowest three detection angles and the highest detection angle deviated significantly from the overall linear relationship and were abandoned for calculating the molecular weights. These results indicate that the GPC separation is based on a size exclusion effect in the elution process of the (6FDA-DMB) PAA in columns. Figure 1c shows the molecular weight distribution curve for the original (6FDA-DMB) PAA.

The calculated molecular parameters of the original (6FDA-DMB) PAA in 0.02 *M* LiBr/0.20 *M* HAc/DMF are listed in Table 1. The mass recovery of the PAA sample was 91.5%, implying there was almost no adsorption between PAA and the chromatographic columns. The *M*_w_ was 247.3 kg/mol, the *M*_n_ was 153.1 kg/mol, the *M*_w_/*M*_n_ was 1.62, the z-average radius of gyration *R*_g,z_ was 29.6 nm, and [*η*] was 161.4 mL/g.

### 3.2. Molecular Parameter Changes of (6FDA-DMB) PAA in Concentrated Solution under Different Storage Conditions

**Figure 2:**
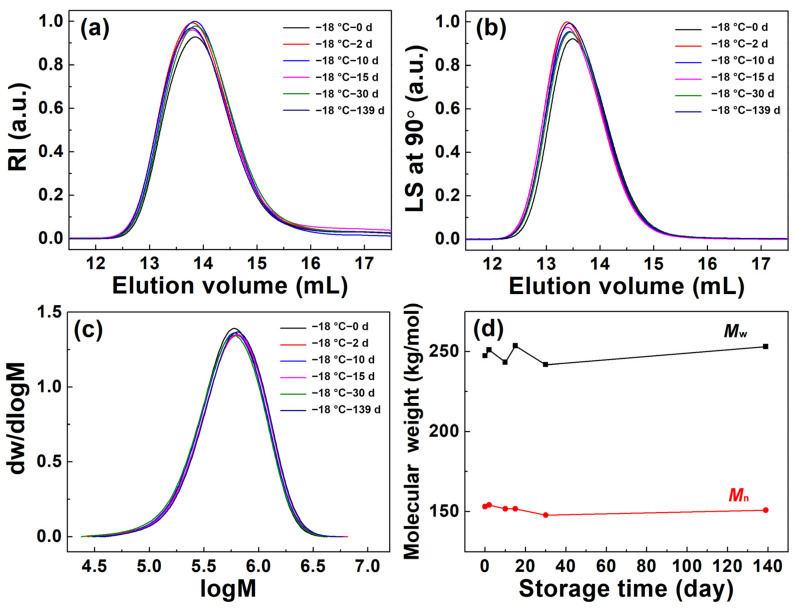
(**a**) RI and (**b**) LS at 90° signals as a function of elution volume, (**c**) molecular weight distribution curves, and (**d**) variation of *M*_w_ and *M*_n_ with storage time for PAA stored at −18 °C. The label −18 °C-0 d represents the original PAA solution, while −18 °C-2 d represents that it was stored at −18 °C for 2 days.

GPC-RI-MALLS-VIS was employed to monitor the changes of molecular parameters such as *M*_w_, *M*_n_, *M*_w_/*M*_n_, *R*_g_, and [*η*], of the (6FDA-DMB) PAA in concentrated solution stored at −18 °C, −12 °C, 4 °C, and 25 °C for different times. Figure 2 shows the elution curves from detectors and the molecular weight distribution of (6FDA-DMB) PAA in the concentrated solution stored at −18 °C for different times. The detailed molecular parameters are listed in Table 2. The mass recoveries of the (6FDA-DMB) PAA samples stored at −18 °C for different days were all more than 90%. During the storage for 139 days, the *M*_w_ varied in the range of 241.8–253.1 kg/mol, and the *M*_n_ varied in the range of 147.7–154.0 kg/mol. The errors for both *M*_w_ and *M*_n_ were within 5%. All other molecular parameters, including molecular weight distribution *M*_w_/*M*_n_, the root-mean-square radius of gyration *R*_g_, and intrinsic viscosity [*η*], changed little. These results show that the molecular parameters of (6FDA-DMB) PAA in a concentrated solution stored at −18 °C remain constant for 139 days.

**Figure 3:**
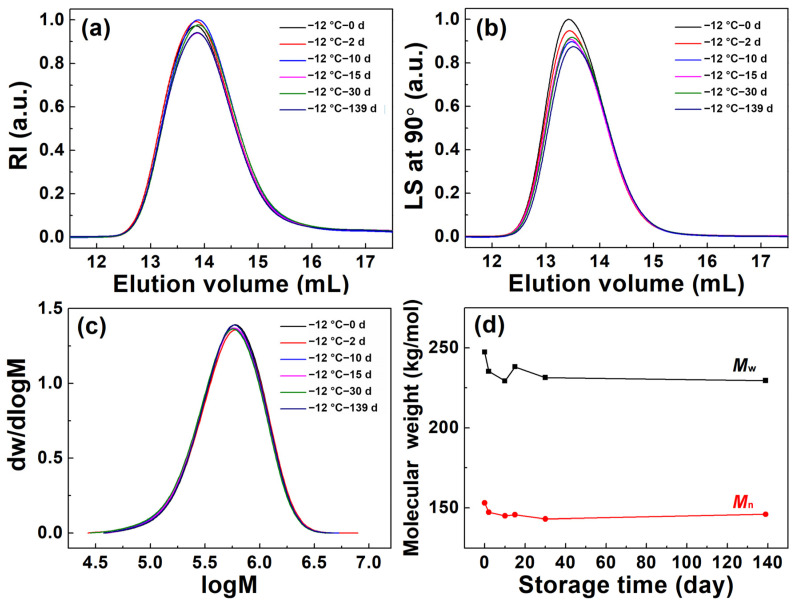
(**a**) RI and (**b**) LS at 90° signals as a function of elution volume, (**c**) molecular weight distribution curves, and (**d**) variation of *M*_w_ and *M*_n_ with storage time for PAA stored at −12 °C. The label −12 °C-0 d represents the original PAA solution, while −12 °C-2 d represents that it was stored at −12 °C for 2 days.

Figure 3 shows the elution curves obtained from RI and LS detectors and the molecular weight distribution of (6FDA-DMB) PAA in a concentrated solution stored at −12 °C for different amounts of days. The calculated molecular parameters are summarized in Table 3. All samples showed a mass recovery higher than 90%, implying that there was almost no adsorption of PAA to the GPC columns. At days 0, 2, 10, 15, 30, and 139, the *M*_w_ was 247.3, 235.3, 229.3, 238.2, 231.4, and 229.5 kg/mol; the *M*_n_ was 153.1, 147.2, 144.9, 145.6, 143.1, and 145.9 kg/mol; the *M*_w_/*M*_n_ was 1.62, 1.60, 1.58, 1.64, 1.62, and 1.57; the *R*_g_ was 29.6, 29.3, 28.6, 29.6, 28.9, and 28.6 nm; and the [*η*] was 161.4, 160.5, 168.9, 176.2, 169.3, and 159.8 mL/g, respectively. Obviously, molecular parameters, including *M*_w_, *M*_n_, and *R*_g_, slightly decrease with the elongation of storage time at −12 °C.

**Figure 4:**
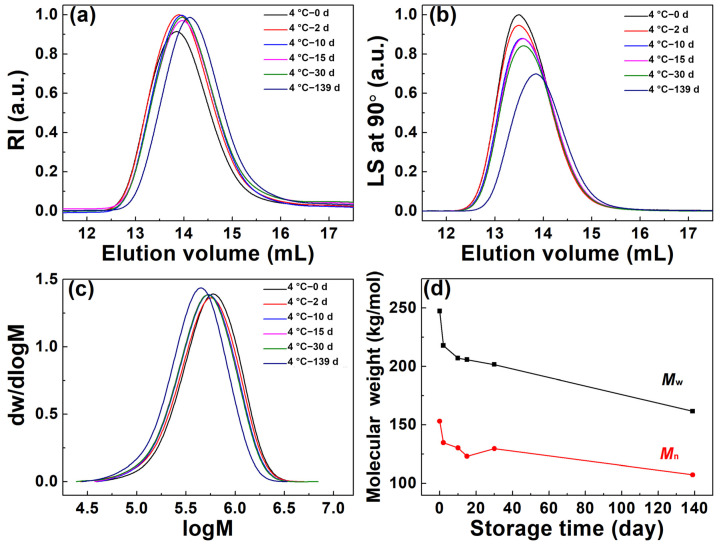
(**a**) RI and (**b**) LS at 90° signals as a function of elution volume, (**c**) molecular weight distribution curves, and (**d**) variation of *M*_w_ and *M*_n_ with storage time for PAA stored at 4 °C. The label 4 °C-0 d represents the original PAA solution, while 4 °C-2 d represents that it was stored at 4 °C for 2 days.

Figure 4 shows the elution curves detected from RI and LS detectors and the corresponding molecular weight distribution of (6FDA-DMB) PAA in a concentrated solution stored at 4 °C for different times. It can be seen that the RI and LS elution curves for different storage times were not coincident and the initial elution volume delayed with the storage time, indicating that the molecular weight of (6FDA-DMB) PAA decreased with the storage time at 4 °C. The calculated molecular parameters are listed in Table 4. The mass recoveries of all samples were above 90%. The *M*_w_ decreased gradually from 247.3 kg/mol to 161.6 kg/mol and the *M*_n_ decreased from 153.1 kg/mol to 107.3 kg/mol when the storage time increased from 0 day to 139 days. A reduction in the polydispersity index *M*_w_/*M*_n_ from 1.62 to 1.51 was observed after the solution was stored for 139 days. The *R*_g_ also showed a decrease from 29.6 nm to 23.4 nm, and the [*η*] decreased from 161.4 mL/g to 127.0 mL/g after 139 days. Evidently, *M*_w_, *M*_n_, *R*_g_, and [*η*] show a gradual decline, and the molecular weight distribution becomes narrower with storage time.

**Figure 5:**
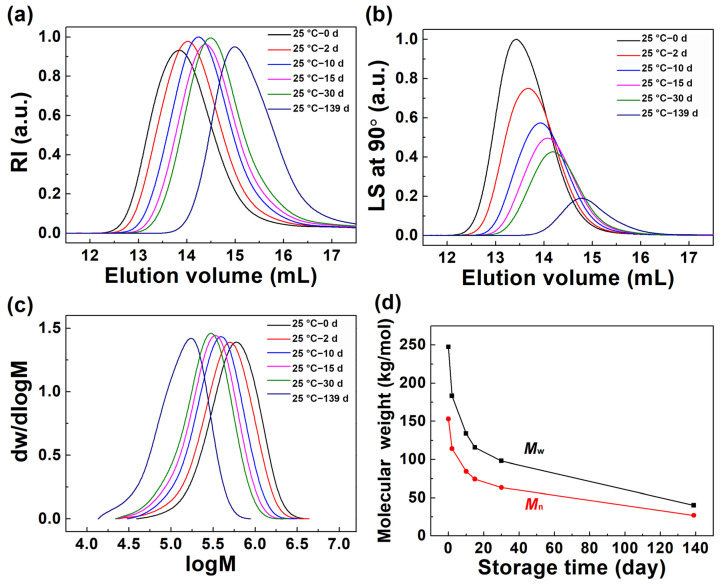
(**a**) RI and (**b**) LS at 90° signals as a function of elution volume, (**c**) molecular weight distribution curves, and (**d**) variation of *M*_w_ and *M*_n_ with storage time for PAA stored at 25 °C. The label 25 °C-0 d represents the original PAA solution, while 25 °C-2 d represents that it was stored at 25 °C for 2 days.

Figure 5 displays the RI and LS signals as a function of elution volume and the molecular weight distribution of (6FDA-DMB) PAA in a concentrated solution stored at 25 °C for different times. Figure 5a,b show that the elution curves shifted to higher elution volume with the storage time, indicating that the molecular weights of (6FDA-DMB) PAA dramatically decreased with the storage time at 25 °C. The obtained molecular parameters are listed in Table 5. PAA samples had no adsorption on the column packings due to a mass recovery higher than 90%. After storage for 139 days, the *M*_w_ decreased from 247.3 kg/mol to 40.1 kg/mol, with a decrease ratio of up to 83.8%, and the *M*_n_ correspondingly decreased from 153.1 kg/mol to 27.0 kg/mol, with a reduction ratio of 82.4%. The narrowing of the molecular weight distribution was indicated by the reduction of *M*_w_/*M*_n_ from 1.62 to 1.49. The *R*_g_ gradually decreased from 29.6 nm, 25.9 nm, 21.5 nm, 20.6 nm, and 18.8 nm to 11.6 nm, and the [*η*] gradually decreased from 161.4 mL/g, 137.5 mL/g, 117.2 mL/g, 105.4 mL/g, and 94.8 mL/g to 51.5 mL/g. The *M*_w_, *M*_n_, *R*_g_, and [*η*] absolutely decreased intensely with storage time at 25 °C, and the molecular weight distribution tended to be narrower. As shown in Figure 5d, the degradation of the polyamic acid was faster in the first 15 days, and the molecular weight was reduced to about half of the initial value; after that, the degradation gradually slowed down. Even though the solvent used in this study was HPLC grade, water was inevitably present. The *o*-carboxylic acid group acted as an intramolecular catalyst for the amide bond cleavage [22]. The resulting anhydrides reacted with trace water in the solvent to form diacids, which were inert toward the polyamic acid-forming reaction. As a result, the molecular parameters, such as *M*_w_, *M*_n_, *R*_g_, and [*η*], decreased with storage time.

**Figure 6:**
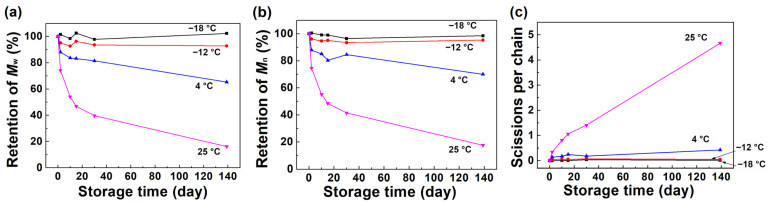
Retention of (**a**) *M*_w_, (**b**) *M*_n_, and (**c**) scissions per chain of (6FDA-DMB) PAA in a concentrated solution stored at −18 °C, −12 °C, 4 °C, and 25 °C for different times.

**Figure 7:**
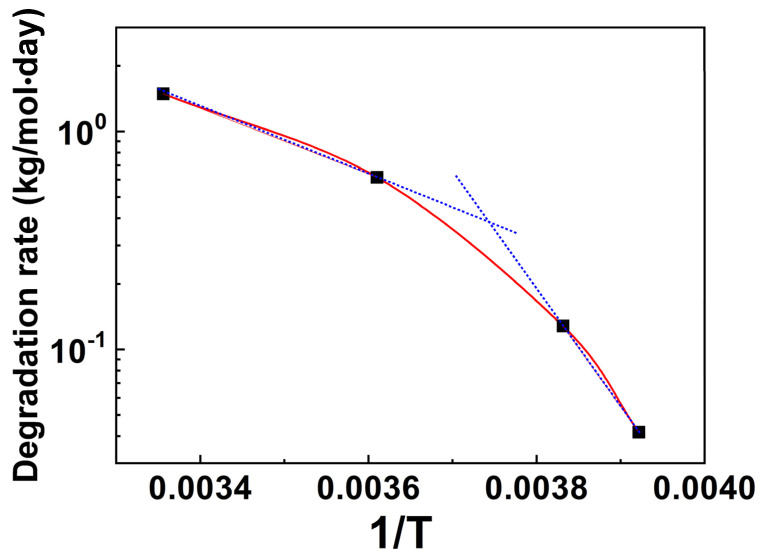
The log (degradation rate) vs. 1/T curve of (6FDA-DMB) PAA in a concentrated solution stored at −18 °C, −12 °C, 4 °C, and 25 °C.

The changes of *M*_w_, *M*_n_, and scissions per chain of (6FDA-DMB) PAA in a concentrated solution stored at −18 °C, −12 °C, 4 °C, and 25 °C for different times are summarized in Figure 6. The retention of *M*_w_ and *M*_n_ were obtained by calculating the percentage of molecular weights at any time to the original molecular weights. The scissions per chain were calculated using (*M*_n,0_ − *M*_n,i_)/*M*_n,i_, where *M*_n,0_ is initial *M*_n_ and *M*_n,i_ is *M*_n_ at any time. The *M*_w_ and *M*_n_ of (6FDA-DMB) PAA remained basically unchanged at −18 °C with storage time, and no chain scission occurred. The *M*_w_ and *M*_n_ dropped slightly at −12 °C with storage time. After storage at −12 °C for 139 days, the reduction ratio of *M*_w_ was 7.2%, while that of *M*_n_ was 4.7%. The scissions per chain were about 0.05, which means that only one out of every 20 chains broke down. The *M*_w_ and *M*_n_ stored at 4 °C were reduced by 34.7% and 30.0%, respectively. Meanwhile, the *R*_g_ and [*η*] also exhibited a decrease of 20.9% and 21.3%, respectively. Those reductions in molecular parameters suggested that PAA was degraded at 4 °C and one out of every 2.5 chains broke down, indicated by the scissions per chain value of 0.43. When the storage temperature increased to 25 °C, all the molecular parameters decreased much more rapidly compared with the other three temperatures. The *M*_w_ and *M*_n_ were found to decrease by 83.8% and 82.4%, respectively, after storage at 25 °C for 139 days. The breakage of amide bonds was about five scissions per chain. The *R*_g_ and [*η*] also showed a pronounced loss of 60.8% and 68.1%, respectively. In addition, the decline rates of *M*_w_ and *M*_n_ were faster in the first 15 days by a drop of 53.2% and 51.4%, respectively. At this point, one break of amide bonds occurred on each chain, and then the chain scission slowed down. It is noteworthy that *M*_w_ decreased faster than *M*_n_ at different temperatures, which resulted in a narrower molecular weight distribution. At –12, 4 and 25 °C, the polydispersity index *M*_w_/*M*_n_ decreased by 3.1%, 6.8%, and 8.0%, respectively, after storage for 139 days. Both percentages of decrease in *M*_w_ and *M*_n_ were the highest at 25 °C, followed by 4, –12, and –18 °C. This revealed that the storage stability of a PAA solution is enhanced at lower temperatures by inhibiting hydrolysis. Reductions in both *M*_w_ and *M*_n_ suggested that the degradation of PAA chains did not follow the reported chain length equilibration, where a reduction in *M*_w_ and almost constant *M*_n_ were observed [14]. The data at four temperatures (−18 °C, −12 °C, 4°C, and 25 °C) and log (degradation rate) vs. 1/T curve were plotted as shown in Figure 7. Here, the degradation rate was defined as *M*_w_ decreased in 139 days (kg/mol∙day), which was calculated from |*M*_w_,_0_ − *M*_w_,_139_|/139. The temperature threshold was about −6°C, at which the dependence of degradation rate on temperature started to change.

### 3.3. Molecular Parameter Change of (6FDA-DMB) PAA in Diluted Solution Stored at 25 °C for Different Times

**Figure 8:**
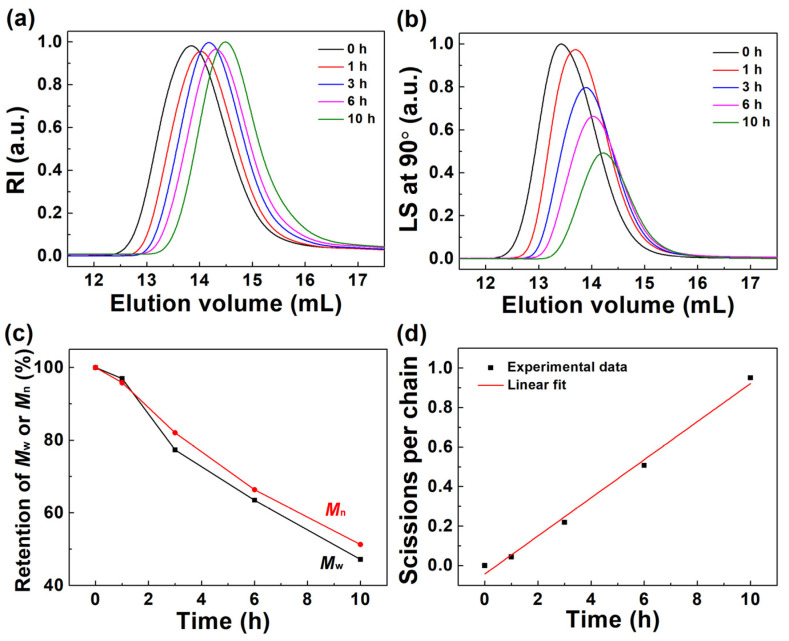
(**a**) RI and (**b**) LS at 90° signals as a function of elution volume. Retention of (**c**) *M*_w_ and *M*_n_ and (**d**) scissions per chain of (6FDA-DMB) PAA in the diluted solution stored at 25 °C for different times.

The 12% PAA solution was diluted to 0.15% solution, and the effect of concentration on PAA stability was investigated in detail. The GPC measurements of PAA in diluted solution were carried out after storage at 25 °C for 0, 1, 3, 6, and 10 h. Figure 8a,b show the elution curves from RI and LS detectors, and the relevant molecular parameters are presented in Table 6. The initial elution volume shifted to larger values with the elongation of storage time, indicating a decrease in molecular weights. The percentages of molecular weight retention are displayed in Figure 8c. The *M*_w_ and *M*_n_ dramatically decreased by 52.8% and 48.7%, respectively, after storage for 10 h. In addition, the [*η*] also significantly decreased by 54.4%. During the first 1.5 h of storage, *M*_w_ and *M*_n_ declined at similar rates, and the molecular weight distribution was almost unchanged. After 1.5 h, *M*_w_ declined faster than *M*_n_, and the molecular weight distribution became narrower. The reduction ratio of *M*_w_/*M*_n_ was 8.0% after being stored for 10 h. Figure 8d indicates that the PAA in the diluted solution was degraded basically linearly over time within 10 h of storage. The amide bonds broke down by about one scission per chain after storage for 10 h. This degradation degree was comparable with that of the concentrated solution stored at the same temperature for 15 days. Therefore, the degradation of PAA in the diluted solution was much more rapid than that in the concentrated one. One reason for this faster degradation of PAA in the diluted solution was that the water ratio introduced by the solvent was much higher than that in concentrated solution. In addition, the molecular chains of (6FDA-DMB) PAA in the diluted solution were more likely to contact with water molecules than those in the concentrated solution, due to the difference in the entanglement of chains [23]. These factors accelerated the hydrolysis of amide bonds and the consequent molecular weight decline of PAA in the diluted solution. Similar to concentrated solutions, the degradation of PAA in the diluted solution did not conform to the chain length equilibration mechanism.

## 4. Conclusions

The solution stability of polyamic acid formed from the condensation reaction of 6FDA and DMB was studied by evaluating the variation of molecular parameters, especially *M*_w_ and *M*_n_, during storage, using GPC-RI-MALLS-VIS. The effects of storage temperature, storage time, and solution concentration on the molecular parameters were systematically investigated.

The stability of PAA in concentrated solution (12%) presented a strong decline with increasing temperature. Specifically, the molecular weights basically remained unchanged at −18 °C for 139 days. When the storage temperature increased from −12 and 4 to 25 °C, the *M*_w_ decreased by 7.2%, 34.7%, and 83.8%, while the *M*_n_ decreased by 4.7%, 30.0%, and 82.4%, respectively, after storage for 139 days. The corresponding scissions per chain increased from 0.05 and 0.43 to 4.67 with the increase of temperature. This dependence of the PAA degradation rate on temperature was caused by faster hydrolysis of amide bonds with trace water in the solvent at high temperatures.

The diluted solution (0.15%) exhibited worse stability than that of the concentrated one stored at the same temperature (25 °C), which was caused by faster degradation due to a higher water ratio and less entanglement of chains. The reduction ratio of *M*_w_ and *M*_n_ were 52.8% and 48.7%, respectively, after storage for 10 h, which was comparable with that of the concentrated solution stored at the same temperature for 15 days. The PAA in the diluted solution showed an almost linear degradation within 10 h of storage at 25 °C. Both reductions in *M*_w_ and *M*_n_ observed in this study suggest that the degradation of PAA does not follow the chain length equilibration mechanism.

In conclusion, storing PAA concentrated solutions in dry and sealed bottles below −18 °C is recommended to obtain a stable PAA solution for at least 4 months. Variations of molecular parameters during storage demonstrated in this study are helpful in tuning PAA solution properties and the consequent performances of PI films or fibers.

## Figures and Tables

**Table 1:** Molecular parameters of original (6FDA-DMB) PAA in 0.02 *M* LiBr/0.20 *M* HAc/DMF, measured by GPC-RI-MALLS-VIS.

Sample	*M*_w_ (kg/mol)	*M*_n_ (kg/mol)	*M*_w_/*M*_n_	*R*_g,z_ (nm)	[*η*] (mL/g)	Recovery (%)
Original PAA	247.3	153.1	1.62	29.6	161.4	91.5

**Table 2:** Molecular parameters of (6FDA-DMB) PAA in a concentrated solution stored at −18 °C for different times, measured by GPC-RI-MALLS-VIS.

Storage Time (day)	*M*_w_ (kg/mol)	*M*_n_ (kg/mol)	*M*_w_/*M*_n_	*R*_g,z_ (nm)	[*η*] (mL/g)	Recovery (%)
0	247.3	153.1	1.62	29.6	161.4	91.5
2	251.0	154.0	1.63	30.4	165.1	99.6
10	243.3	151.7	1.60	29.4	173.5	98.3
15	253.6	151.6	1.67	30.3	176.2	99.8
30	241.8	147.7	1.64	29.4	171.0	98.9
139	253.1	150.8	1.68	30.7	169.5	98.7

**Table 3:** Molecular parameters of (6FDA-DMB) PAA in a concentrated solution stored at −12 °C for different times, measured by GPC-RI-MALLS-VIS.

Storage Time (day)	*M*_w_ (kg/mol)	*M*_n_ (kg/mol)	*M*_w_/*M*_n_	*R*_g,z_ (nm)	[*η*] (mL/g)	Recovery (%)
0	247.3	153.1	1.62	29.6	161.4	91.5
2	235.3	147.2	1.60	29.3	160.5	99.7
10	229.3	144.9	1.58	28.6	168.9	95.0
15	238.2	145.6	1.64	29.6	176.2	96.0
30	231.4	143.1	1.62	28.9	169.3	98.6
139	229.5	145.9	1.57	28.6	159.8	93.9

**Table 4:** Molecular parameters of (6FDA-DMB) PAA in a concentrated solution stored at 4 °C for different times, measured by GPC-RI-MALLS-VIS.

Storage Time (day)	*M*_w_ (kg/mol)	*M*_n_ (kg/mol)	*M*_w_/*M*_n_	*R*_g,z_ (nm)	[*η*] (mL/g)	Recovery (%)
0	247.3	153.1	1.62	29.6	161.4	91.5
2	217.9	134.7	1.62	28.0	153.6	99.2
10	207.0	130.4	1.59	27.7	156.0	97.9
15	205.8	123.0	1.67	27.3	158.4	98.8
30	201.6	129.6	1.56	27.1	151.9	99.3
139	161.6	107.3	1.51	23.4	127.0	95.4

**Table 5:** Molecular parameters of (6FDA-DMB) PAA in a concentrated solution stored at 25 °C for different times, measured by GPC-RI-MALLS-VIS.

Storage Time (day)	*M*_w_ (kg/mol)	*M*_n_ (kg/mol)	*M*_w_/*M*_n_	*R*_g,z_ (nm)	[*η*] (mL/g)	Recovery (%)
0	247.3	153.1	1.62	29.6	161.4	91.5
2	183.2	114.2	1.60	25.9	137.5	99.9
10	133.7	84.6	1.58	21.5	117.2	98.3
15	115.8	74.4	1.56	20.6	105.4	99.5
30	98.1	63.5	1.54	18.8	94.8	97.5
139	40.1	27.0	1.49	11.6	51.5	99.6

**Table 6:** Molecular parameters of (6FDA-DMB) PAA in the diluted solution stored at 25 °C for different times, measured by GPC-RI-MALLS-VIS.

Storage Time (h)	*M*_w_ (kg/mol)	*M*_n_ (kg/mol)	*M*_w_/*M*_n_	[*η*] (mL/g)	Recovery (%)
0	247.3	153.1	1.62	161.4	91.5
1	239.8	146.7	1.64	116.6	94.3
3	191.3	125.6	1.52	102.5	91.7
6	157.0	101.6	1.55	91.7	91.1
10	116.7	78.5	1.49	73.6	91.9

## Data Availability

The data presented in this study are available on request from the corresponding author.
